# Examining a stepped-care telehealth program for parents of young children with autism: a proof-of-concept trial

**DOI:** 10.1186/s13229-021-00443-9

**Published:** 2021-05-08

**Authors:** Allison L. Wainer, Zachary E. Arnold, Caroline Leonczyk, Latha Valluripalli Soorya

**Affiliations:** 1 Department of Psychiatry and Behavioral Sciences, Rush University Medical Center, 1645 W. Jackson Blvd., Suite 603, Chicago, IL 60612 USA; 2 Department of Psychology, University of Alabama At Birmingham, 1300 University Blvd., Birmingham, AL 35233 USA

**Keywords:** Autism spectrum disorder, Telehealth, Online RIT, Reciprocal imitation training, Stepped-care, Digital intervention

## Abstract

**Background:**

Intervention during the first years of life for children with autism spectrum disorder (ASD) may have the strongest impact on long-term brain development and functioning. Yet, barriers such as a shortage of trained professionals contribute to significant delays in service. The goal of this proof-of-concept study was to explore strategies that support timely and equitable deployment of ASD-specific interventions.

**Methods:**

This 15-week, randomized proof-of-concept study explored the acceptability of a digital parent mediated intervention online reciprocal imitation training (RIT; a naturalistic developmental behavioral intervention) and compared it to a treatment as usual (TAU) control on parent and child outcomes. Eligible children were between 18 and 60 months, met the cutoff for ASD on the Autism Diagnostic Observation Schedule-2nd Edition and demonstrate significant social imitation deficits. Primary outcomes include the acceptability of RIT (Scale of Treatment Perceptions) and the feasibility of the Online RIT digital intervention (online RIT attributes). Secondary outcomes included parent fidelity (RIT parent fidelity form) and parental self-efficacy (Early Intervention Parenting Self-Efficacy Scale). Exploratory outcome measures included child social communication (Social Communication Checklist), child imitation skills (Unstructured Imitation Assessment), and family quality of life (Beach Center Family Quality of Life Scale).

**Results:**

Twenty participants were randomized in a 1:1 fashion. The acceptability and feasibility of RIT and the Online RIT digital intervention were rated highly. Among the secondary outcomes, there were significant group differences in parent fidelity (*p* < .001) and self-efficacy (*p* = .029). On exploratory outcomes, there were group differences in child social communication (*p* = .048). There were no significant group differences in imitation ability (*p* = .05) or family quality of life (*p* = .22).

**Limitations:**

There are several limitations with this study, including the small sample size as well as lack of data on enactment and website engagement. This study was not able to address questions related to which variables predict program engagement and treatment response, which will be critical for determining which families may benefit from such a stepped-care delivery model.

**Conclusions:**

Overall, the Online RIT program delivered in a stepped-care format shows strong acceptability and holds promise as an innovative delivery model.

*Trial registration* ClinicalTrials.gov, NCT04467073. Registered 10 July 2020- Retrospectively registered, https://clinicaltrials.gov/ct2/show/NCT04467073

## Background

There are clear benefits to specific early interventions for young children with and at-risk for autism spectrum disorder (ASD) [1]. Yet, limited availability of services results in many children “missing out” on intervention during the key developmental period proposed to have the most powerful effect on gene expression, brain development and behavior [2]. The discrepancy between service need and access is compounded for those in underserved communities where children receive even fewer interventions and at later ages [3]. Commonly cited barriers to care include transportation issues, limited funding, and a shortage of well-trained professionals, all of which contribute to significant waitlists and delays in care [4]. Research examining strategies to directly address these barriers and support deployment of ASD-specific interventions in a timely, equitable and efficient manner is a top priority [5].

### Naturalistic developmental behavioral interventions (NDBIs)

There is growing evidence for Naturalistic Developmental Behavioral Interventions (NDBIs), a category of interventions that merge applied behavioral and developmental sciences, for improving social communication and developmental outcomes in young children with and at-risk for ASD [6]. While each NDBI has specific features, they are characterized by commonalities in terms of the nature of learning targets (e.g., focus on building a social learning infrastructure), learning contexts (e.g., teaching within naturalistic contexts), and development-enhancing strategies. NDBIs share evidence-based strategies such as use of a three-part contingency, child-initiated teaching episodes, environmental arrangement, and natural reinforcement (see 7 for a full list of shared strategies). Best practice indicates that delivery of NDBIs should be initiated as early as possible, include a parent component, and concurrently address underlying core deficits and teach new skills [6, 7].

Comprehensive NDBIs, such as the Early Start Denver Model (ESDM; [8]) target a number of broad functional outcomes and therefore are often intensive in terms of resources, time, and duration, and rely on highly trained interdisciplinary specialists [9]. The importance of comprehensive programs should not be understated; however, the complex nature of these programs may hinder access, adoption and implementation—especially in community settings [10]. In particular, families who have access to traditional comprehensive interventions vastly underutilize the hours they are allotted [11]. Additionally, recent estimates indicate an average time-lag of roughly 3 years between diagnosis and receipt of early intensive behavioral intervention in the community [12]. Further, the younger a child at diagnosis, the greater the time-lag to service access, suggesting a significant gap in care during a period when the child and family may be particularly vulnerable [12].

Given the current services landscape, it is critical to consider additional models such as focused NDBIs, which may be particularly well suited to serve as a “bridge” or supplement to more comprehensive approaches. Focused interventions teach a specific skill or set of skills, are often less complex and intensive, and hold promise for more efficient dissemination and adoption across formats (e.g., parent mediated intervention) and settings (e.g., early childhood classrooms, home-based IDEA Part C services). Research suggests a number of focused NDBIs, such as Reciprocal Imitation Training (RIT) and Joint Attention Symbolic Play Engagement and Regulation (JASPER), can improve specific social communication skills at a relatively low intensity and when implemented by caregivers and other key members of a child’s community [13–16]. Taken together, focused NDBIs are particularly well-suited for use when examining innovative delivery models to provide care to families without or with limited access to evidence-based services.

### Telehealth

Telehealth, or the ability to provide long-distance health care and education, has been one of the most rapidly growing fields of research and clinical care in the last decade. In 2019, 90% of adults in the United States reported using the internet, 81% report owning a smartphone, and approximately 78% of homes had broadband internet connection [17]. Given the intuitive appeal of telehealth, this delivery model has already been adopted in community settings. Prior to the COVID-19 pandemic, two thirds of U.S. hospitals employ some kind of telehealth technology, one third of states have enacted telehealth parity laws, and nearly all state Medicaid programs cover at least some form of telehealth [18]. In 2012, only 13 states had incorporated telehealth into the Part C Early Intervention system [19]); this number has grown dramatically over the last decade, and most recently during the global COVID-19 pandemic, with nearly all states launching initiatives to support statewide implementation of telehealth-based Early Intervention services [20]. The scientific data and stakeholder excitement around telehealth suggests that it is poised to serve as a viable alternative or supplement to traditional service delivery models with potential to deliver care in an effective, acceptable, and cost-efficient manner [21].

Telehealth can be used to deliver intervention in a variety of formats. For example, there is a growing emphasis on the use of digital interventions or self-directed applications (e.g., smart phone apps) and websites to deliver evidence-based intervention such as cognitive behavioral therapy (CBT) for common psychiatric conditions such as depression [22, 23]. Similarly, earlier telehealth early intervention research explored self-directed digital interventions to deliver parent training in NDBIs [24, 25]. While digital interventions provide greater flexibility for user engagement, reduce costs, and facilitate large scale dissemination, limitations to this approach must be considered. Specifically, parents using self-directed digital intervention programs are more likely to report barriers to participation (such as time), less likely engage with and complete program elements, and less likely to report gains in child skills [26, 27]. Research has also examined the use of videoconferencing or “real-time”/synchronous telehealth, to deliver instruction and parent coaching to support skill acquisition in caregivers of children with ASD [28–31]. Despite the promise of these data, remote parent coaching alone still does not address the significant barrier of a shortage of trained professionals in community settings.

Importantly, digital interventions and remote parent coaching approaches can be integrated for more effective service delivery. For example, a parent could access digital tools (in the form of a website or game) to learn the intervention content at their own time at pace, and then connect remotely with a professional to get feedback on their use of the strategies and help with problem solving and planning. Ingersoll and colleagues compared use of an interactive website alone to the website plus remote coaching and found both groups showed gains from pre- to post-intervention [26]. However, parents in the remote-coaching condition improved more in their intervention fidelity and increased positive perceptions of their child, and the children in that condition showed greater gains in social skills.

Although there is limited understanding of the specific variables predicting response to treatment, these data support the notion that tailoring of both digital tools (e.g., interactive website) and coaching approaches should be explored to enhance learning and application of skill from such hybrid telehealth programs [32]. Equally as important is the close consideration of personalized telehealth models to maximize reach and efficiency of service delivery.

### Adaptive interventions

Given the modest evidence for the “one size fits all” telehealth approach, an examination of individualized models for promising NDBIs is warranted. Adaptive interventions allow for variation in intervention intensity as a function of individual or environmental characteristics and treatment response [33]. Adaptive interventions may be especially fitting when there are high levels of heterogeneity in treatment response and a need to consider a cost–benefit trade-off between intervention intensity and available resources [34]. Given the current state of ASD research and practice, there is a strong rationale for exploration of adaptive telehealth NDBIs.

Stepped-care is a model of adaptive intervention which offers less intensive intervention as a first-line treatment, and more intensive care only when clinically indicated. Decisions about who receives “stepped up” care are data-driven in that they are informed by closely monitoring patient outcomes using what are referred to as tailoring variables to determine if and when additional care is indicated. Two of the main indicators of success in parent-mediated interventions are parent fidelity and self-efficacy. Parent fidelity (i.e., the proper implementation of an intervention) has been instrumental for positive increases in child outcomes across several NDBIs [35]. Parent self-efficacy (i.e., parental belief in their capabilities) is thought to serve a vital role in maximizing early intervention outcomes [36].

Stepped-care models have been designed for the delivery of interventions for eating disorders, substance abuse, anxiety, depression and childhood trauma, and data indicate it is an effective, acceptable and cost-efficient model, particularly for brief interventions [37]. Investigators have suggested that stepped-care may be an especially promising delivery model for targeted early interventions for ASD [2] but research has not yet examined a stepped-care parent mediated intervention for ASD.

### Current study

Online RIT is an interactive website introducing Reciprocal Imitation Training (RIT), an NDBI focused on enhancing social imitation [27]. RIT uses a naturalistic behavioral approach to teach object and gesture imitation to young children with ASD within a play-based context. The efficacy of RIT has been demonstrated through a small randomized control trial [13, 14] and several single-subject design studies [38, 39], as well as in independent replications [40–42]. Prior research also suggests that parents can be taught to effectively use RIT with their children in person [15], and two single-subject design studies detail the development and feasibility testing of Online RIT plus therapist assistance [25, 27]. In Study 1, parents used the website and received in-vivo coaching; children showed corresponding improvements in imitation [25]. In Study 2, parents used the website with remote coaching; parents improved in RIT fidelity across study phases, and concurrent improvements in child imitation were observed. All families received remote coaching, but 2/5 achieved the fidelity threshold after the website alone [27]. These preliminary data suggest Online RIT may serve as an ideal platform for examining the potential of individualized telehealth delivery formats, such as stepped-care.

This proof-of-concept study addresses the gap in the current literature and represents an important next step in digital intervention development by examining a stepped-care version of Online RIT. Although not frequently employed in behavioral intervention research, a proof-of-concept study is warranted given the significant financial and resource investment associated with developing and trialing digital interventions. Indeed, proof-of-concept studies are increasingly common early in the lifecycle of digital interventions, particularly in the field of behavioral health [43]. Furthermore, proof-of-concept studies can be used to inform decisions about whether to proceeded with further investment in the tool and larger more expensive studies [44, 45]. As such, the goal of the current study was to determine the acceptability and feasibility of a stepped-care format of Online RIT and to explore initial differences in critical outcomes such as parent fidelity and self-efficacy, when compared to treatment as usual.

## Methods

### Participants

Twenty families of a child with ASD were recruited for the current study via clinician referrals, community partner referrals and recruitment postings on social media. To be eligible for screening, children had to be between 18 and 60 months, have a diagnosis of ASD or significant concerns of ASD, and have parent-reported imitation deficits. Children of parents who were non-English speaking or who were actively participating in other parent training programs were excluded. Families were not charged for study assessments or intervention and they received $40 in amazon.com gift cards for participation.

### Study design

The Institutional Review Board (IRB) at Rush University Medical Center (RUMC) (IRB 15,100,203) approved this 15-week randomized controlled trial (RCT) comparing a stepped-care model of Online RIT to treatment as usual (TAU) condition. Participants signed informed consent at the in-person screening/baseline visit, before any data collection began. Participants completed baseline and post-intervention behavioral assessments at RUMC, while the rest of the data collection and study participation occurred remotely. Questionnaires were collected electronically via Qualtrics, and home-based parent–child interactions were recorded from video conferences using Vidyo (see below). Participants were randomly assigned to one of two conditions using a computerized randomization program.

### Eligibility and sample characteristics

**Fig. 1:**
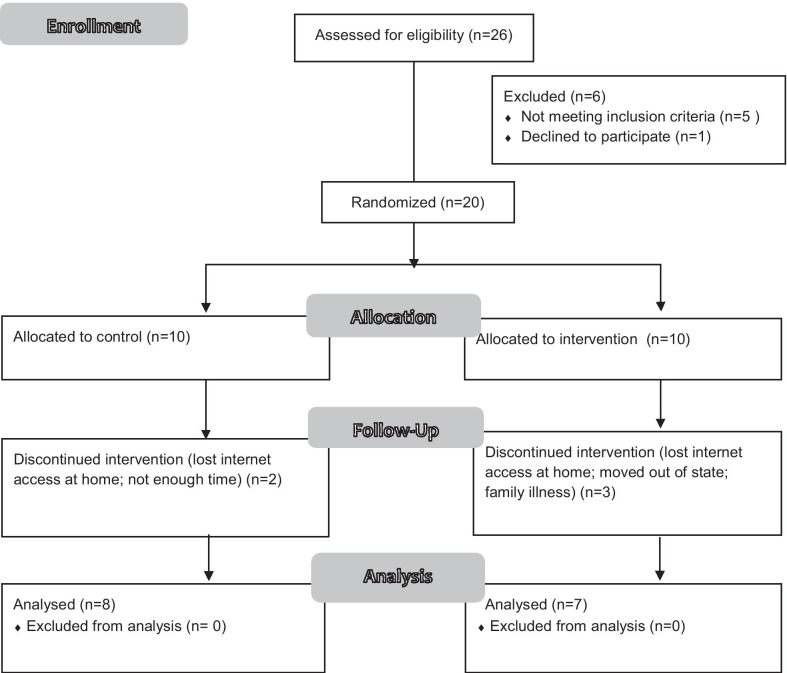
CONSORT diagram

To be eligible for participation in the study, children had to meet the cutoff for ASD on the Autism Diagnostic Observation Schedule-2 Edition [46], and demonstrate significant social imitation deficits (< 50%) on the Unstructured Imitation Assessment (UIA, [14]). Children were also administered the Mullen Scales of Early Learning (Mullen, [47]) to provide an estimate of nonverbal and expressive language levels. See the study CONSORT diagram (Fig. 1) and Table 1 for participant demographic information.

**Table 1:** Participant demographic information

Characteristic	Intervention (*n* = 10)	Control (*n* = 10)
*Child demographics*		
Male (*n*)	8	6
Child age in months (M,SD)	40.10 (10.41)	35.40 (11.09)
Nonverbal age equivalent in months (M,SD)	18.70 (6.68)	22.67 (8.41)
Expressive language age equivalent in months (M,SD)	9.50 (4.14)	14.13 (10.36)
Child race/ethnicity (*n*)		
Asian	1	0
Black	4	0
Caucasian, non-Hispanic	0	5
Hispanic/Latino	4	4
Multiracial	1	1
Weekly intervention hours (M, SD)	7.60 (10.61)	4.73 (9.95)
Weekly intervention hours (median, range)	3.50, 2.00–36.00	1.7, 0.00–32.75
Speech therapy	1.00, 1.00–2.00	1.00, 0.00–2.00
Occupational therapy	1.00, 1.00–2.00	0.00, 0.00–2.00
Applied behavior analysis	0.00, 0.00–30.00	0.00, 0.00–30.00
Physical therapy	0.00, 0.00–2.00	0.00, n/a
Developmental therapy	0.00, 0.00–1.00	0.00, 0.00–1.50
*Parent/family demographics*		
Female	10	10
Parent education		
Some high school	1	1
High school degree	2	0
Some college/specialized training	3	3
4-Year college degree	0	4
Graduate degree	4	1
Employed outside the home	6	4
Parental marital status		
Married-living with partner	4	5
Single-living with partner	4	1
Single-living alone	2	2
Divorced or separated	0	1
Computer use and literacy		
Very comfortable with computers	8	7
Very comfortable with internet	9	9
Daily use of computer at home	10	9
More than 5 times each day on the internet	6	5
More than 7 h per week on the internet	5	5

### Measures

#### Participant characterization

##### Demographics

A demographics form used in prior trials [26] was completed by parents and included information about the child, participating parent, and family structure.

##### Mullen Scales of Early Learning (Mullen; [47])

The MSEL was administered to provide an index of the child’s developmental level at intake. Specifically, Visual Reception age equivalents were used to estimate nonverbal mental age and Expressive Language age equivalents were used to estimate child expressive language age. The MSEL has been shown to have strong construct, convergent, and divergent validity in children with and without ASD [48].

##### Computer-Email-Web (CEW) Fluency Scale [49]

The CEW Fluency Scale is a self-report measure designed to assess an individual’s fluency with the computer, email and the web. For the purposes of the current study, 5-items were used to characterize participant familiarity and comfort with computer and internet technology (see Table 1). The CEW has well established construct validity, convergent validity, and reliability [50].

##### Services questionniare

The *Services Questionnaire* asks parents to indicate the intervention services a child received in the past and the services the child is current receiving, including the number of weekly hours received for each service (e.g., speech therapy, occupational therapy). Total weekly hours of services were summed for a total score for each participant.

#### Primary outcome: acceptability and feasibility

##### Scale of treatment perceptions (STP; [51])

The STP is a measure of treatment acceptability targeting skill building interventions, particularly for children with ASD. The STP was adapted to include language specifically referencing RIT, resulting in a 24 item questionnaire which provides an index of the perceived effectiveness of RIT, the fit between RIT and the family, and the safety of RIT. Participants rate the extent to which they agree with the statements from *Strongly Disagree* (1) to *Strongly Agree* (7). The STP has demonstrated appropriate internal consistency, a stable factor structure, and divergent validity [51].

##### Online RIT attributes

This scale was adapted from Moore and Benbasat’s [52] instrument to measure the perceptions of adopting an information technology innovation. This brief adapted version (18 items) has been used in intervention studies similar to the current work [53]. Participants indicate level of agreement from *Strongly Disagree* (1) to *Strongly Agree* (7). This adapted scale provides domain scores mapping onto four critical characteristics of innovations [54]: observability, complexity (with higher scores reflecting less complexity), acceptability, relative advantage. The original instrument was found to have acceptable convergent validity, divergent validity, and reliability [52].

#### Secondary outcomes: parent measures

##### RIT parent fidelity form (RIT-PFF; [27])

Trained observers scored the parent–child interactions for parent fidelity of the RIT intervention techniques using the RIT fidelity form (see [27] for behavioral definitions). Parent behavior was rated from 1 (low) to 5 (high) across six domains: Contingent Imitation, Linguistic Mapping, Modeling, Prompting, Reinforcement and Pacing. The last four domains were averaged to derive a Prompting Sequence score. Ratings on Contingent Imitation, Linguistic Mapping and Prompting Sequence were averaged for an Overall Fidelity Score. Raters were blind to participant condition and time point. Ten percent were double coded to ensure interrater reliability of 80% or higher.

##### Early Intervention Parenting Self-Efficacy Scale (EIPSES; [55])

The EIPSES is a 20-item parent questionnaire designed to measure parenting efficacy within the context of early intervention (e.g., “when my child shows improvement, it is because I am able to make a difference in my child’s development”). Participants rate the extent to which they agree with statements from *Strongly Disagree* (1) to *Strongly Agree* (7). The EIPSES provides two index scores (Parent Outcomes Expectations and Parent Competence) and an overall score. For the purposes of the current study, the EIPSES overall score was used for data analysis. The EIPSES has demonstrated strong reliability and construct validity [55].

#### Exploratory outcomes: child and family measures

##### Social communication checklist (SCC; [56])

The SCC is a 47-item checklist completed by parents to indicate if a child uses a specific social communication skill *Rarely/Not Yet* (1), *Sometimes, but not consistently* (2), or *Usually, at least 75% of the time* (3). Scores in the areas of social engagement, language/communication and imitation/play can be derived and then summed for an SCC Total Score. Psychometric analyses have demonstrated that the SCC is reliable, sensitive to measuring change after an intervention, and strongly related to other measures of social-communication functioning [57].

##### Unstructured imitation assessment (UIA; [14])

The UIA was used to measure child social imitation. It is a standardized assessment that evaluates spontaneous imitation of actions with objects and gestures during play. The examiner provides 20 different imitation bids (10 object, 10 gesture). Each bid is repeated three times. Child responses are rated as “0” none, “1” partial, or “2” full. The highest score for each imitation bid is summed for an overall UIA score. The UIA was coded by raters blind to participant condition and time point for the current study. Thirty percent were double coded to ensure interrater reliability of 80% or higher.

##### Beach Center Family Quality of Life Scale (FQOL Scale; [58])

The FQOL Scale is a 25-item self-report measure designed to assess family interaction, parenting, emotional well-being, physical/maternal well-being, and disability-related supports. Participants rate the extent to which they are satisfied with these various aspects of family interaction and experience from *Very Dissatisfied* (1) to *Very Satisfied* (5). An overall Total Score was calculated by averaging all items. The FQOL has been found to have satisfactory construct validity, internal reliability, and convergent validity [59].

### Intervention and service delivery platforms

Online RIT is a digital intervention in the form of an interactive website that was developed to teach RIT to parents of young children with or at-risk for ASD. Program development was guided by the technology acceptance model, media richness theory (i.e., which technologies best reduce uncertainty and equivocality), and principles of instructional design [60–62]. A collaborative and iterative development process with pilot participants was employed to ensure acceptability and usability. Online RIT is hosted on a unique URL owned and managed by RUMC, requires a unique username and password to log in, and is consistent with “best practices” in terms of safeguards to ensure website security. The program is mobile device and computer compatible.

Online RIT presents RIT techniques in four sequential learning modules: (1) *Setting Up For Success* (selecting activities, antecedent controls, scheduling practice time, ensuring a support system); (2) *Imitating your Child* (contingent imitation, imitating the child’s vocalizations, gestures, body movements and play with toys); (3) *Describing Play* (linguistic mapping, using simple and descriptive language at or slightly above the child’s linguistic level); (4) *Teaching Object Imitation* (using modeling, prompting, reinforcement to teach a target skill, and pacing the interaction). Each learning module includes an instructional video, quiz, interactive exercises, and at-home planning and reflection. The website also includes a video library, Frequently Asked Questions, downloadable visual aids, links to relevant external resources, and a customizable “dashboard” that allows users to track their individualized goals and the amount of time they have spent working on their goals (e.g., practice log).

Parent coaching sessions were held remotely using Vidyo, which provides secure bidirectional audio and video conferencing capability along with advanced capabilities such as content/screen sharing, video streaming, far end camera control, encryption, DTMF controls and more. Vidyo meets HIPAA privacy standards and has built-in security management (e.g., SSL certificates, private key management, and HTTPS all FIPS 140-2 compliance). Participants in the current study downloaded an app to their smartphone, tablet or computer to allow for seamless videoconferencing.

### Study procedures

#### Screening/baseline

Participants attended one-to-two days of testing at RUMC to complete participant characterization assessments and outcomes. Immediately after screening/baseline visits, participants engaged in a remote video recorded 10-min parent–child interaction.

#### Randomization

A computerized randomization program was used to determine treatment condition assignment following screening/baseline assessments. Participants were enrolled on a 1–1 schedule to Online RIT or TAU.

#### Study conditions

##### Stepped-care online RIT

**Fig. 2:**
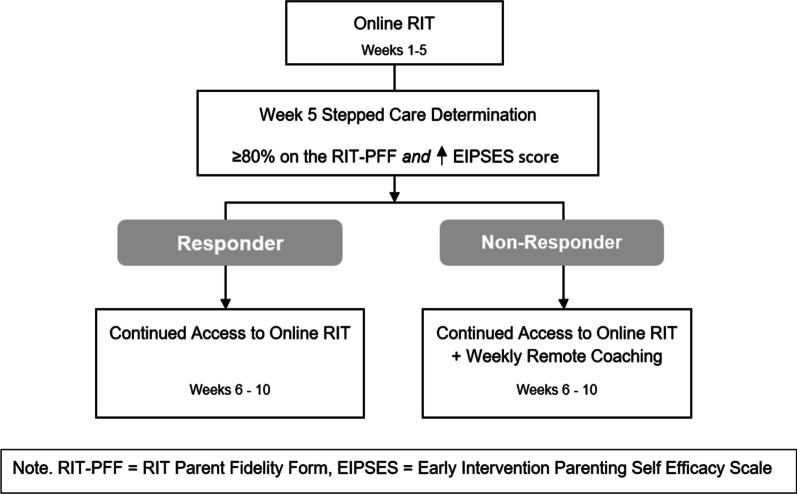
Stepped-care procedures

Parents randomized to Online RIT completed the four modules over a period of 5 weeks (~ 1 per week, 1 week to practice). Prior research on parent-mediated intervention in RIT and related NDBIs suggests improvements in, and pivotal mediating roles for, parent fidelity and parent empowerment/self-efficacy [35, 63]. As such, these two variables were selected as tailoring variables for this stepped-care model. Fidelity (RIT-PFF) and self-efficacy (EIPSES) at 5 weeks were used to determine which participants were in need of a “step up” in care, in the form of remote parent coaching. See the Online RIT Fig. 2 for stepped-care procedures.

Parents who demonstrated ≥ 80% on the RIT-PFF, and who reported gains on the EIPSES continued to have access to Online RIT and practiced on their own for the next 5 weeks but did not receive any remote coaching. Parents who demonstrated < 80% fidelity on the RIT-PFF and/or who didn’t report increases in the EIPSES were directed into coaching. Coaching involved videoconferences once per week (wks. 6–10) with a parent coach (first author), and followed the occupational performance coaching model which assists parents in creating an environment that is more suited for themselves and their child to succeed [64]. Sessions included review of successes and challenges, parent practice with feedback, problem solving, and planning.

##### Treatment as usual (TAU)

Participants in the TAU group were asked to try to keep the child’s current interventions stable for the length of the trial. If changes in family needs required a change in therapies, families were asked to notify the research team. These participants were given the opportunity to engage in the stepped-care format of Online RIT after the post-intervention data collection time point; however, their data was included exclusively in TAU analyses.

*Post-intervention (15 weeks)* Participants returned to RUMC for post-intervention assessments of parent functioning and child social communication. Fidelity was coded from the remote parent–child interactions immediately after the clinic visit.

### Data analysis

Data on families who completed the study were analyzed using IBM SPSS Statistics, Version 22. Initial analyses included examination of baseline group equivalence using independent sample t-tests. Data were inspected for violations of the assumptions for each test prior to running it and were analyzed accordingly. Descriptive statistics were examined to characterize the acceptability of the stepped-care model of Online RIT and the related technology. Analysis of Covariance (ANCOVA) was used to evaluate treatment outcomes by comparing outcome measures at 15 weeks between the Online RIT and TAU groups, after controlling for T1 scores.

## Results

Descriptive statistics for demographic characteristics and baseline outcome variables are provided in Table 1. In general, participants were receiving few intervention hours in the community with 80% reporting ≤ 4 h per week; however, the observed difference in total intervention hours between the two groups was driven by the fact that two participants in the intervention condition were receiving intensive applied behavior analysis (ABA) at 16 and 36 h per week respectively, while only one participant in the TAU group participated in intensive ABA at 30 h per week.

### Acceptability and feasibility

Descriptive statistics were used to characterize the acceptability of the stepped-care model of Online RIT and the related technology as rated by participants who completed the program (Table 2). Responses on the STP indicated strong acceptability of RIT as a skill building intervention. Participants rated RIT as very safe and effective, and endorsed items such as it being a “good fit” for their child and family. Average scores across the Online RIT Attributes scale suggest that as a technological innovation, Online RIT has strong potential for adoption. Responses suggest that Online RIT is acceptable, relatively easy to use, and families supported items such that the intervention is easy to “see” and “explain to others” as to why Online RIT is user-friendly. Further, responses suggest that while Online RIT is consistent with other services a child receives, it also offers unique information that parents find helpful.

**Table 2:** Acceptability of online RIT

Acceptability outcomes	M (SD)
*Scale of treatment perceptions*	
RIT safety	6.67 (0.39)
RIT effectiveness	6.61 (0.34)
RIT family fit	5.81 (1.08)
*Online RIT attributes*	
Online RIT relative advantage	6.00 (0.57)
Online RIT acceptability	6.36 (0.54)
Online RIT limited complexity	6.00 (0.74)
Online RIT observability	6.50 (0.60)

Range of scores: (1–7)

### Parent outcome measures

Parent outcome data did not violate assumptions for ANCOVA. Thus, ANCOVAs were run to determine the effect of the stepped-care model of Online RIT on post-intervention parent variables after controlling for baseline scores on these same variables (Table 3). After adjusting for baseline scores, there were significant differences in post-intervention outcomes between groups on ratings of parent fidelity, *F*(1,12) = 44.59, *p* < 0.001, Cohen’s *d* = 3.86, and EIPSES scores, *F*(1,12) = 6.185, *p* = 0.029, Cohen’s *d* = 1.44. Post hoc analyses were performed with a Bonferroni adjustment and indicated that post-intervention parent fidelity ratings were significantly greater for Online RIT relative to TAU (*M*_diff_ = 2.56, 95% CI [1.72,3.39], *p* < 0.001). Post-intervention EIPSES scores were also significantly greater for Online RIT vs. TAU (*M*_diff_ = 9.86, 95% CI [1.22, 18.50], *p* = 0.029).

**Table 3:** Online RIT secondary and exploratory outcomes

	Intervention		Control		ANCOVA			Pairwise	
Variable	Adjusted M	SE	Adjusted M	SE	F	p	Cohen’s D	Mean difference	95% CI
*Parent outcomes*									
RIT fidelity	4.33	0.27	1.77	0.26	44.59	> .001	3.86	2.56	1.72, 3.39
EIPSES	118.19	2.88	108.33	2.70	6.19	.029	1.44	9.86	1.22, 18.50
*Child outcomes*									
SCC total	146.61	5.72	129.34	5.35	4.84	.048	1.27	17.27	0.16, 34.37
UIA	8.54	1.33	4.40	1.24	4.75	.050	1.26	–	–
*Family outcomes*									
FQOL total	108.02	2.72	103.20	2.55	1.68	.220	0.75	–	–

EIPSES, Early Intervention Parenting Self Efficacy Scale; UIA, Unstructured Imitation Assessment; SCC, Social Communication Checklist; FQOL, Beach Center Family Quality of Life Scale

Individual differences in response to Online RIT were observed. Only one parent was considered a full responder after the website alone. This parent met the parent fidelity threshold (i.e., ≥ 80% on the RIT-PFF) and demonstrated increases in self-efficacy (i.e., reported gains on the EIPSES) and therefore did not receive coaching. Two parents who met criteria for fidelity of RIT reported slight declines in self-efficacy from baseline to post-website, and therefore received coaching. Four additional parents received coaching as they did not meet RIT fidelity threshold. After receiving coaching, five of the six parents achieved fidelity and increased ratings of self-efficacy from baseline to post-intervention.

### Child and family outcome measures

Child and family outcome data did not violate assumptions for ANCOVA. Thus, ANCOVAs were run to determine the effect of Online RIT on post-intervention child variables after controlling for baseline scores on these same variables (Table 3). After adjusting for baseline scores, there were significant differences in post-intervention outcomes between groups on SCC Total scores *F*(1,12) = 4.863, *p* = 0.048, Cohen’s *d* = 1.27. Post hoc analyses were performed with a Bonferroni adjustment. Post-intervention SCC Total scores were significantly higher in the Online RIT group relative to TAU (*M*_diff_ = 17.267, 95% CI [0.160,34.374], *p* = 0.048). No stasistically significant differences were found between groups on the UIA (*F*(1,12) = 4.75, *p* = 0.050) or the FQOL (*F*(1,12) = 1.68), *p* = 0.220). Acknowleding challenges with estimating effect sizes from small studies, effect sizes favor RIT for both UIA (Cohen’s *d* = 1.26) and FQOL (Cohen’s *d* = 0.75). Given the observed effect size favoring the UIA, the Leeds Reliable Change Index [65] was used to assess for significant changes on an individual level in imitation performance across the sample. Results suggested that 3 out of the 7 children in Online RIT demonstrated reliable improvement in performance on the UIA while no children from the control group showed similar reliable improvement. Further, one participant from the control group demonstrated a reliable decline in UIA performance. None of the children in the Online RIT group showed reliable decline in UIA performance.

## Discussion

The current proof-of-concept study is the first to evaluate a stepped-care telehealth parent training program for delivering ASD-specific intervention. Families of young children with or at-risk for ASD experience significant uncertainty and stress during the period that they are waiting for an evaluation and initiation of comprehensive early intervention services [66]. In addition, many children are “missing out” on intervention during critical developmental periods [2]. Thus, the development and evaluation of innovative interventions and delivery models, such as Online RIT, with potential to deliver timely, equitable and cost-efficient ASD-specific intervention to young children is critical.

RIT, as a skill building intervention, was experienced as safe, effective and a good fit for families. The Online RIT program was rated highly across characteristics known to be associated with more successful dissemination of novel interventions such as acceptability, relative advantage, observability and lack of complexity. Perceived ease of use and perceived usefulness are particularly important in the context of technological innovations [60]. Qualitative feedback about the ease of use and usefulness of Online RIT included “Ease of logging in at any time, ease of pausing and then coming back to complete tasks, a great library of videos to learn from,” “The [Online] RIT website allowed me to view real-life examples of techniques. It's easier and entertaining to watch the techniques than to read about them,” and “It's a great way to see what other people are doing and how to apply it with my own son!” These data suggest strong potential for the deployment of RIT across diverse settings and service delivery formats including via the Online RIT program.

This is the first study to examine the potential of a stepped-care model for an ASD telehealth intervention. Even within this small sample, differences in parent learning and experience were observed. For example, only one parent was considered a full responder and therefore did not require coaching. This parent achieved the fidelity threshold and showed increases in self-efficacy after using the website alone. Our iterative process and user-centered framework provides a data-driven approach to better meet diverse user needs [67]. For example, one route is to use rapidly evolving technological innovations to improve engagement and individualized support through the digital intervention itself. A participant suggested “I recommend a schedule to complete the lessons. A way of tracking when I'm done with the homework and saving it also informing me when it's time to do the reflection. Case in point, my first lesson I did everything at once without practicing the skill first.” Building in personalized recommendations for how and when to use the digital intervention could not only enhance learning but allow users to feel more competent and successful along the way. Consistent with best practices in digital intervention development [67] *real time* multi-dimensional assessment of participant experience (e.g., website usage, ecological momentary assessment (EMA; [68]) of self-efficacy) would also allow for immediate identification of participants who would benefit from the addition of a coach, thereby reducing the time lag time between need and access.

Another important goal of this proof-of-concept study was to explore differences in intervention outcomes between participants in the Online RIT group and those in TAU. After adjusting for baseline scores, parents in the intervention condition showed larger improvements in parent fidelity of RIT and parent self-efficacy. While it is not surprising that parents who received Online RIT learned the strategies better than parents who did not receive the program, these data support the notion that Online RIT can be an effective model for parents to learn ASD-specific intervention strategies and change their behavior accordingly during interactions with their young children at home. All but one parent in the Online RIT condition eventually implemented RIT with fidelity, suggesting that with the right supports RIT is a relatively easy NDBI to learn and use. Indeed, prior research has found that despite overall improvements in skill after digital interventions and remote coaching in more complex NDBIs, many parents still do not achieve the fidelity threshold [32]. As noted above, there is a rich opportunity to capitalize upon technological innovations to build more engaging and supportive digital intervention programs. Concurrently, it is important to note that these predetermined fidelity thresholds are also a legacy from lab-based efficacy studies, and the necessity or impact of achieving the fidelity threshold remains unclear in parent-mediated intervention studies.

Consistent with research from parent mediated NDBIs, data from the current study suggest a meaningful relationship between Online RIT and parent self-efficacy [69]. Self-efficacy is an important driver of individual behavior change [70], with higher self-efficacy related to a greater likelihood of embracing and sustaining novel behaviors, even when faced with obstacles [71]. It is possible that parents who feel more efficacious in interactions with their child with ASD may engage with their child more often, have higher expectations for their child’s behavior, and/or more effectively advocate for their child’s needs, all of which may contribute to improved parent and child level outcomes, especially during a time of uncertainty and need. Interestingly, two parents in the Online RIT group who were observed to be implementing RIT with fidelity after using the website alone reported experiencing declines in self-efficacy during that same period. Again, these patterns point to the need to incorporate more adaptive features into the digital intervention platform, as well as the need to identify certain subsets of individuals who could benefit from coaching and at earlier stages, to enhance parenting efficacy in response to such a program.

No significant differences between the two groups on broad family quality of life at post-intervention were observed. This is an area that is often not directly measured within early intervention for ASD trials, as primary outcomes tend to be related to the child (e.g., social communication, developmental functioning) or to the parents’ use of the intervention techniques (e.g., fidelity of the intervention [72]). However, enhancing a family’s capacity to problem solve, advocate and seek out supports are considered critical outcomes of early intervention programs [67]. There is potential to build features into the digital intervention program that could have a stronger impact on broad family quality of life; as one participant explained Online RIT could house “tips/videos of parents embedding RIT into regular everyday routines, tips and resources for using RIT with siblings around and in community play groups with peers, success stories from parents to encourage other parents, inspiration and play ideas from parents, a forum for parents to exchange ideas and motivate each other and discuss how they fit it into their busy lives.”

After adjusting for baseline scores, children in the intervention condition had improved scores on parent report of overall social communication skills. Although significant differences between the two groups were not observed on the UIA, the effect size favored the intervention condition with three of these children showing reliable improvement in their UIA performance. While the small sample size could help account for the lack of significant group differences on the UIA, it is also important to note that standardized clinician-administered social communication assessments as primary endpoints command a high threshold that may not be sensitive to subtle markers of change in early social communication skills [73]. Thus, it is possible that a certain amount of time and intensity of intervention may be necessary for generalized social communication gains to be observed on such standardized clinical assessments [73].

The extent to which parents actually practiced and implemented these strategies with their children day-to-day is unknown. This metric, often referred to as enactment or dose, is extremely challenging to measure and is historically under-reported in studies of parent mediated interventions for ASD and related conditions [35, 74]. In the current study, a text message approach to collecting this data was trialed. First, the study team text messaged three times per week (“Have you used RIT with your child today? Y/N”; if Y “For about how many minutes did you use RIT today?”). Although families initially responded, the response rate dropped to zero after two weeks. Parents reported that these texts were “too frequent,” so the rate was reduced to once a week, but the similar response rate was observed. The rapid rate of technological innovation means that certain features, such as a pop-up calendar that emerges when a user signs into the website, are now possible in a way that they were not when the site was first developed. A critical area for future research will be to work with families and other key stakeholders to determine acceptable, effective and feasible strategies for collecting this critical enactment data.

### Limitations

Several limitations to the current study have been noted in the discussion section including the small sample size, lack of enactment data, and a lack website engagement data. Given the nature of the proof-of-concept trial, we were not able to offer a robust test of treatment effectiveness, although preliminary data on child imitation and family functioning are promising. In addition, the rapid shift to digital healthcare during the pandemic has highlighted both the potential and challenges of telehealth care [75]. It is acknowledged that telehealth and digital interventions may not be universally acceptable and/or effective for all families. The extent to which critical family characteristics such as cultural background, race, and ethnicity, impact access to and engagement with the Online RIT program could not answered in the current work. In addition, the extent to which parent motivational and cognitive variables, as well as child skills, moderate treatment effectiveness remains unknown. Such work will be critical as we incorporate technology allowing for more individualization of the digital intervention and coaching approach, as well as for determining a priori who would benefit from such a delivery model and who may be better served through a different format. Finally, despite random assignment, the two groups were unbalanced on certain background characteristics such race and ethnicity (with greater racial and ethnic diversity in the Online RIT intervention group relative to TAU) and average weekly intervention hours (with the Online RIT intervention group recieving, on average, more hours each week than TAU).

## Conclusions and future directions

With the limitations above noted, the current proof-of-concept study still serves as an important initial step in exploring the feasibility of a stepped-care format of Online RIT. We found that parents enjoyed the intervention content and website, that they were able to learn and use RIT with their children as a result of the program, and that they had ideas and suggestions for further improving the digital intervention user experience. Taken together, these results compel additional investment in the digital intervention program and offer clear next steps in the larger scale evaluation of this innovative intervention approach. For example, we selected parent fidelity of RIT and parent self-efficacy as tailoring variables in the current model based on theoretical rationale, but by design we were not able to explicitly test whether these are the “right” tailoring variables or the extent to which different levels of care predicted specific response to treatment across outcomes of interest. In addition to identifying appropriate tailoring variables, a related critical next step of this work is to understand how participant characteristics, including socioeconomic status and demographic characteristics, impact engagement with and completion of digital interventions. Moreover, future research will empirically test the efficiency and effectiveness of possible active components of the intervention and to understand the extent to which these components can be adjusted to provide more individualized intervention. Theoretically informed pilot data from this trial are critical for use of novel research strategies such as Multiphase Optimization Strategy (MOST; [33]) which has been posited as an alternative to the traditional randomized control/confirmatory trial in the development and evaluation of telehealth and digital interventions. Indeed, planned future research with Online RIT will capitalize upon such innovative research designs to create and evaluate an optimized adaptive telehealth intervention.

## Data Availability

The datasets used and analyzed for the current study are available from the corresponding author upon reasonable request.

## Abbreviations

ANCOVA: Analysis of covariance
ASD: Autism spectrum disorder
CBT: Cognitive behavioral therapy
CEW: Computer-email-web
EIPSES: Early Intervention Parenting Self-Efficacy Scale
ESDM: Early start denver model
FQOL: Family quality of life
IRB: Institutional Review Board
JASPER: Joint Attention Symbolic Play Engagement and Regulation
MOST: Multiphase optimization strategy
MSEL: Mullen Scales of Early Learning
NDBIs: Naturalistic developmental behavioral interventions
RCT: Randomized controlled trial
RIT: Reciprocal imitation training
RIT-PFF: RIT parent fidelity form
RUMC: Rush University Medical Center
SCC: Social Communication Checklist
STP: Scale of treatment perceptions
TAU: Treatment as usual
UIA: Unstructured imitation assessment
